# The Association between Gut Microbiome Diversity and Composition and Heat Tolerance in Cattle

**DOI:** 10.3390/microorganisms10081672

**Published:** 2022-08-19

**Authors:** Xiaohui Zhang, Ke Cui, Xiaobo Wen, Lianbin Li, Xiangchun Yu, Boling Li, Haichao Lin, Hongxuan He, Fengyang Wang

**Affiliations:** 1 College of Animal Science and Technology, Hainan University, Haikou 570228, China; 2 Promotion Station of Animal Husbandry Technology of Hainan Province, Haikou 572999, China; 3 Institute of Zoology, Chinese Academy of Sciences, Beijing 100101, China

**Keywords:** fecal microbiome, heat tolerance, regional climates, cattle

## Abstract

Cattle are raised around the world and are frequently exposed to heat stress, whether in tropical countries or in regions with temperate climates. It is universally acknowledged that compared to those in temperate areas, the cattle breeds developed in tropical and subtropical areas have better heat tolerance. However, the underlying mechanism of heat tolerance has not been fully studied, especially from the perspective of intestinal microbiomics. The present study collected fecal samples of cattle from four representative climatic regions of China, namely, the mesotemperate (HLJ), warm temperate (SD), subtropical (HK), and tropical (SS) regions. Then, the feces were analyzed using high-throughput 16S rRNA sequencing. The results showed that with increasing climatic temperature from HLJ to SS, the abundance of *Firmicutes* increased, accompanied by an increasing *Firmicutes* to *Bacteroidota* ratio. *Proteobacteria* showed a trend of reduction from HLJ to SS. *Patescibacteria*, *Chloroflexi*, and *Actinobacteriota* were particularly highest in SS for adapting to the tropical environment. The microbial phenotype in the tropics was characterized by an increase in Gram-positive bacteria and a decrease in Gram-negative bacteria, aerobic bacteria, and the forming of_biofilms. Consistently, the functional abundances of organismal systems and metabolism were decreased to reduce the material and energy demands in a hot environment. Genetic information processing and information storage and processing may be how gut flora deals with hot conditions. The present study revealed the differences in the structure and function of gut microbes of cattle from mesotemperate to tropical climates and provided an important reference for future research on the mechanism of heat tolerance regulated by the gut microbiota and a potential microbiota-based target to alleviate heat stress.

## 1. Introduction

Livestock provide a fundamental source of food to people, accounting for 18% of calories and more than 25% of protein globally [1]. However, heat stress resulting from high environmental temperatures and relative humidity often exhibits prolonged and deleterious effects on the productivity, fecundity, and immunity of livestock, including life threats [2,3]. The adverse influence is especially prominent in summer and is exacerbated by global warming [1]. Cattle are raised all over the world and are also frequently exposed to heat stress, whether in tropical countries or in regions with temperate climates [4]. Almost all effects of heat stress are similar between dairy and beef cattle [5].

Compared to breeds developed in temperate areas, there are some breeds of cattle developed in tropical and subtropical areas that have better heat tolerance. For instance, zebu cattle developed in China (Yunnan Province) and India are regarded as breeds with a stronger thermoresistance ability [6]. Leiqiong cattle are also a highly heat-resistant breed and are raised in the Leizhou Peninsula and Hainan Island, which are characterized by a subtropical/tropical climate [7,8]. Interestingly, it is said that people in the Ming dynasty took some Leiqiong cattle to Sansha city in China, a typical tropical region, and the cattle became well adapted to the high-heat climate and have lived there for more than three hundred years so far.

Many studies have revealed that acute or short-term heat stress induces self-protective and adaptive responses in cattle, including behavioral responses, physiological responses (such as respiratory rate, heart rate, and sweating rate), neuro-endocrine responses (such as the hypothalamic–pituitary–adrenal axis), and molecular responses (heat shock protein expression) to restore homeostasis [9,10]. However, the mechanism of adaptability improvement brought by environmental domestication under long-term heat stress remains to be studied.

Recently, a lot of attention has been given to the gastrointestinal microbiome and its metabolites, which have a crucial role in the maintenance of host homeostasis in both humans and animals [11,12]. It is particularly important for cattle that depend on their gastrointestinal microbiota to digest and convert the indigestible plant mass that makes up their diet into absorbable nutrients necessary for host physiological health [13]. Previous studies have shown that varying types of stress could alter the microbial composition, such as social stress in mice, heat stress in laying hens, and weaning stress in dairy cows, thus further modifying the brain-gut axis function for stress and behavioral responses [11]. Moreover, gut microbes can regulate animal intake via the connection of muramyl dipeptide from gut bacteria and Nod2 in inhibitory neurons to modulate appetite and body temperature, which shows us that the influences of gut microbes are not only affecting short-term modulation but were also affecting enduring living habits [14]. The same is true in disease regulation. There is evidence that the community of microbes in the gastrointestinal tract is an important determinant of inflammation, obesity, type 2 diabetes, arterial stiffness, and hypertension, all of which are chronic processes [15].

Direct studies highlight that the gut microbiota has adaptive potential for the environment in humans and animals. A survey of the gut microbiota of Tibetans from six regions with altitudes ranging from 2800 to 4500 m found that altitude (hypoxia exposure) had a positive correlation with the abundance of *Faecalibacterium*, *Bacteroides* and *Bifidobacterium* but a negative correlation with the proportion of *Ruminococcaceae*, *Prevotella*, and *Lachnospiraseae* [16]. Chinese rhesus macaques living in Tibet had gut microbiota with higher environmental information processing and organismal systems than those in other geographical populations [17]. The alteration of gut microbiota by the long-term supplementation with steam-exploded pine particles reduced the adverse effects of heat stress in chickens [18].

Together, these findings inspired us to study the correlation between the temperature in different geographical regions and gut microorganisms in cattle. The present study selected cattle from four different climatic zones (temperature zones) in China to analyze their fecal characteristics of microbial diversity and composition, identified microbial markers in line with regional temperature features, and provided the necessary information for explaining the heat tolerance of cattle in tropical regions and a research basis for future efforts to develop microbiota-based countermeasures that alleviate heat stress.

## 2. Materials and Methods

### 2.1. Animals

Cattle aged 4–6 years old (A), 1.5–2 years old (B), and 6 months to 1 year old (C) from Daqing city of Heilongjiang Province (HLJ), Qingdao city of Shandong Province (SD), Haikou city of Hainan Province (HK), and Sansha city of Hainan Province (SS) in China were researched in the present study. The climate of these areas is characterized by mesotemperate (daily mean temperature at 7.3 ± 14.4 °C), warm temperate, subtropical, and tropical climates, respectively. Between two adjacent climatic zones, the difference in accumulated temperatures is greater than 10 °C (i.e., the sum of the daily mean temperature of the days when the daily mean temperature exceeds 10 °C) in a year [19]. Cattle in the corresponding areas above suffer different challenges from the temperature. In other words, frequent heat stress from high temperatures is the main feature in Sansha and Haikou, while cold stress is an unavoidable environmental adversity in Heilongjiang. Moreover, the temperature in Shandong is moderate. The herd of Leiqiong cattle (about 400 cattle) in Sansha live freely in isolation on an island and only feed on local weeds and shrub leaves. Leiqiong cattle from Haikou are raised artificially in the teaching practice base of Hainan University and fed the silage corn, dry haulm, local weeds, and concentrated feed. The breeds from Shandong and Heilongjiang are Simmental and Holstein, respectively, and are kept on local private ranches, eating mainly silage corn, alfalfa, and concentrated feed. The general compositions of concentrated feed include 56% corn, 6% soybean meal, 8% cottonseed meal, 1.2% vegetable oil, 13% rapeseed dregs, 12.5% wheat bran, 1.3% CaHPO_4_, 0.5% NaCl, 0.5% NaHCO_3_, and 1.0% premix. The proportion of each component is slightly adjusted at different growth stages in this study. The population size of the cattle in the practice base and private farms is 300–600.

### 2.2. Fecal Sampling

Three fresh fecal samples (50 g each) were manually and randomly collected from different healthy animal individuals of each age bracket from the corresponding population. The cattle feces of cattle from different ages in Sansha were sampled under the guidance of an experienced veterinarian. However, owing to the difficulty of tracking closely, only one feces sample from cattle aged 1.5–2 years old in Sansha was collected (Table 1). Fecal materials were transported in a low-temperature environment as soon as possible and stored at −20 °C until analysis.

### 2.3. 16S rRNA Sequencing

The fecal microbiome composition was determined by 16S rRNA gene sequencing as previously described [20]. Briefly, after total DNA was extracted from fecal samples, the V4 region of the 16S rRNA gene was amplified using PCR to build qualified libraries, which were sequenced with an Illumina NovaSeq 6000 by Biomarker Technologies Corporation (Beijing, China).

### 2.4. Sequence Data Processing

QIIME2 2020.6 was used to process and analyze 16S sequencing data [21]. Samples were demultiplexed using q2-demux and denoised using Dada2 to generate amplicon sequence variants (ASVs) [22]. Taxonomy was assigned to sequences against the Silva reference database using QIIME software [23]. Then, R language tools were used to draw the community structure map of the sample at different taxonomic levels.

### 2.5. Correlation Analysis and Differential Feature Selection

The correlation between climatic locations on the beta diversity of the fecal microbiota was determined using QIIME software through the method of principal coordinate analyses (PCoA) based on the abundance Jaccard algorithm [24,25]. Differentially abundant bacteria were determined using line discriminant analysis (LDA) effect size (LEfSe) for identifying biomarkers with significant differences. Functional predictions were performed using PICRUSt [26]. BugBase was used to infer microbiological phenotypes [27].

### 2.6. Statistical Analysis

PERMANOVA and ANOSIM non-parametric tests were used to further evaluate the significance of the observed partitions among samples grouped by geographical origin. Differences in the microbial data between groups were evaluated at the levels of phylum and genus through the rank sum test (software package version: scipy 0.14.1). Significance was defined as *p* < 0.05. Data are represented as mean ± standard deviation.

## 3. Results

### 3.1. Quality Assessment of Sequencing Data and ASV Analysis

Raw reads in a range of 79,717 to 80,363 (80,045.0588 ± 175.020335) were obtained from each sample, and after quality control, clean reads in a range of 79,177 to 80,057 (79,635.705882 ± 219.257912) were further analyzed, with a length of 400–440 base pairs. A total of 363–605 ASVs were received for each sample, and a total of 2883 ASVs were detected (Table S1 and Figure S1).

### 3.2. Taxonomy Analysis

**Figure 1:**
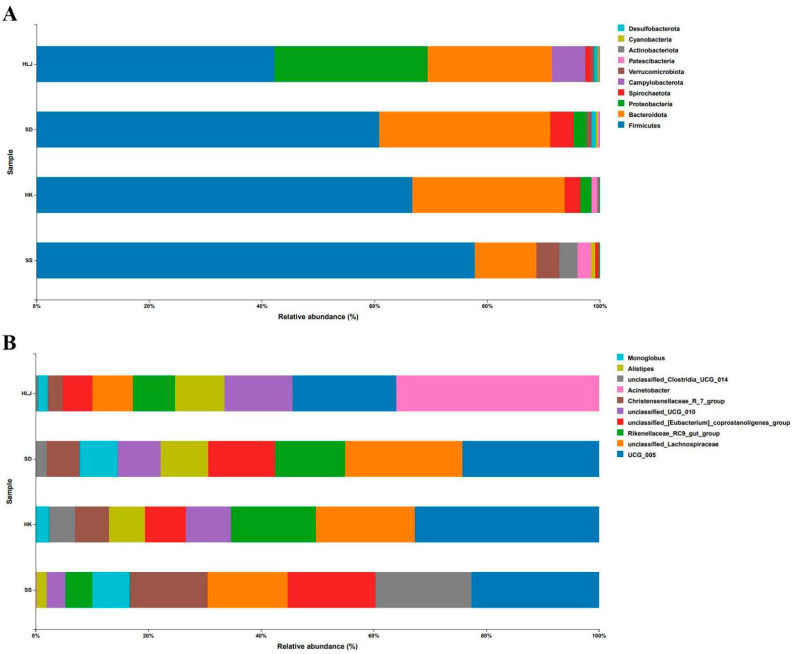
Histogram of the top 10 microbiota in each sampling location at the levels of phylum (**A**) and genus (**B**).

All information of taxonomy and their (relative) abundance could be found in Tables S2 and S3. As shown in Figure 1A, at the phylum level, *Firmicutes* was the most dominant phylum, accounting for 42.40%, 60.60%, 66.71%, and 77.37% of all sequences in the HLJ, SD, HK, and SS groups, respectively. *Bacteroidota* ranked second in relative abundance, accounting for 22.13%, 30.22%, 27.07%, and 10.89% in the mentioned groups. The proportion of *Proteobacteria* was 26.89% in HLJ, 23.37% in SD, 1.99% in HK, and 0.28% in SS. The relative abundance of *Spirochaetota* was 0.99% in the HLJ group and 4.10%, 2.69%, and 0.57% in the SD, HK, and SS groups, respectively. *Verrucomicrobia* accounted for 4.04% in the SS group and no more than 1% in the other three groups. The proportion of *Campylobacterota* was up to 5.82% in HLJ and 0.03% in SD, but 0% in HK and SS.

At the genus level, as displayed in Figure 1B, the proportion of *UCG_005* was 7.44%, 12.26%, 19.64%, and 11.59% in the HLJ, SD, HK, and SS groups, respectively. The relative abundance of *unclassified Lachnospiraceae* accounted for 2.93% in HLJ, whereas it was 10.38%, 10.30%, and 7.29% in SD, HK, and SS, respectively. The proportion of the *Rikenellaceae_RC9_gut_group* in the corresponding sampled areas was 3.03%, 6.25%, 8.97%, and 2.48%, respectively. The proportion of *unclassified_[Eubacterium]_coprostanoligenes_group* in HLJ, SD, HK and SS was 2.09%, 5.99%, 4.38%, and 7.99%, respectively, while that of *unclassified_UCG_010* was 4.89%, 3.90%, 4.83%, and 1.70%. In addition, *Christensenellaceae_R_7_group*, *unclassified_Clostridia_UCG_014*, *Alistipes*, and *Monoglobus* also accounted for a certain proportion in all groups, while *Acinetobacter* accounted for only HLJ.

### 3.3. No Significant Difference Was Observed in Microbiota Diversity among the Cattle from the Same Location

Next, we analyzed the changing profile of the fecal microbiota with increasing age. For the cattle belonging to the same region, at the levels of phylum and genus shown in Figures S2 and S3, the microbial composition and its corresponding proportion did not show statistically significant differences among the cattle in the three age brackets. Therefore, all cattle from the same area were grouped together for subsequent analysis.

### 3.4. The Microbiota Diversity of Cattle Displayed an Obvious Association with Geographical and Climatic Features

**Figure 2:**
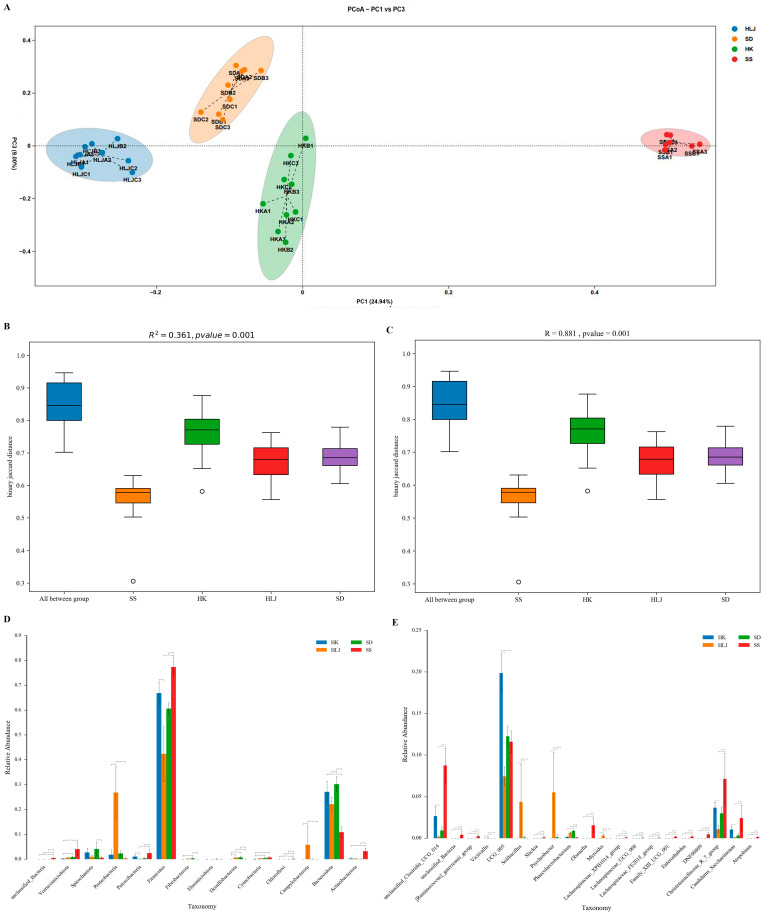
The microbiota diversity of cattle display an evident association with geographical position. (**A**) Beta diversity analysis shows the sequential association of microbiota diversity among the sampled locations in the dimension of PC1. (**B**,**C**) the distant separations among the four locations are confirmed using the methods of PERMANOVA and ANOSIM, respectively. (**D**,**E**) Kruskal–Wallis rank sum test further analyzes the statistical significance of microbiome partitions above at the phylum and genus levels. Data are expressed as mean ± standard deviation. * *p* < 0.05, ** *p* < 0.01.

Beta diversity analysis was used to compare the differences among different groups in terms of microbiota diversity. The results revealed that samples from the different locations showed very distant separation, whereas samples from the same area were rather clustered (Figure 2A). In the dimension of PC1, samples clustered according to the region were remarkably separated from each other in the order of geographical location, namely, HLJ, SD, HK, and SS (from mesotemperate to tropical, in other words, from cold areas to hot places). In particular, the fecal microbial structure of cattle from Sansha was significantly distant from those of the other geographical groups. These huge distances among the four locations were further confirmed by PERMANOVA and ANOSIM tests (Figure 2B,C), and Kruskal–Wallis rank sum test at the phylum and genus levels (Figure 2D,E).

### 3.5. There Were Biomarkers to Match the Geographical and Climatic Characteristics

**Figure 3:**
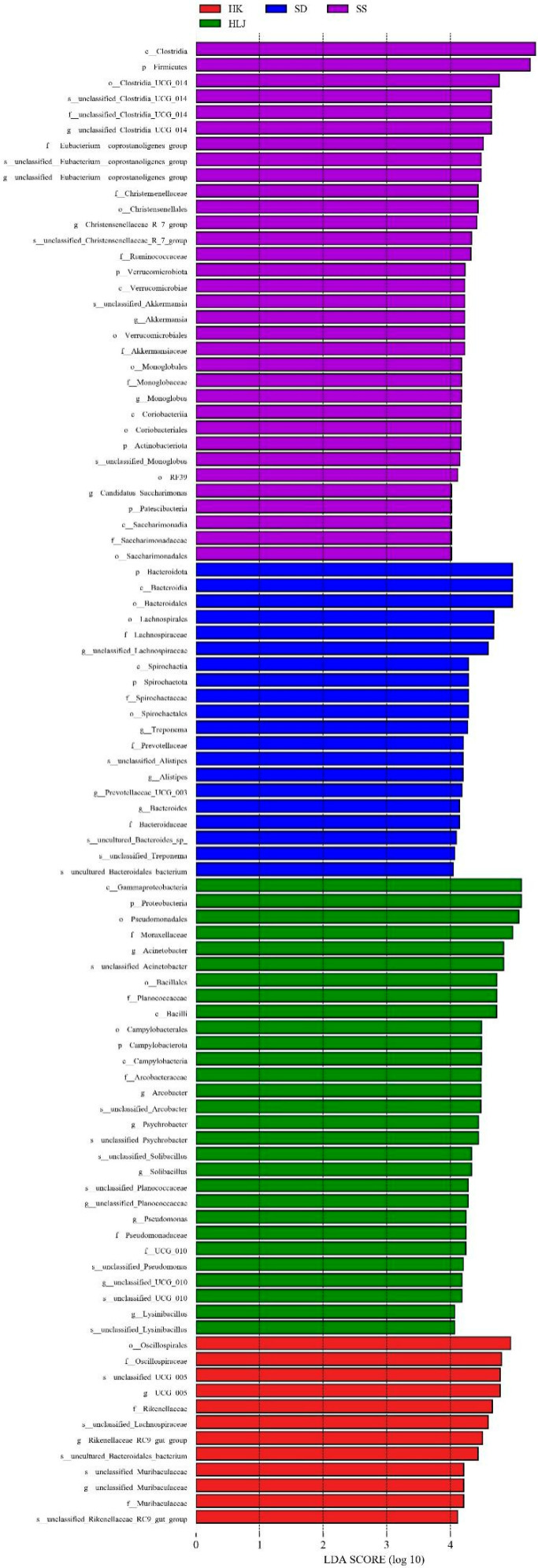
LEfSe analysis shows 94 biomarkers of fecal microbiota from four sampled locations. Histogram of LDA scores represent the differences in the proportions of fecal microbiota. Taxa meeting an LDA significant threshold of >4 are shown.

Using LEfSe analysis, a total of 94 biomarkers from four groups were considered statistically significant (33 in the SS, 20 in the SD, 29 in the HLJ, and 12 in the HK groups), as shown in Figure 1, Figure 3, Figures S4 and S5. At the phylum level, the *Firmicutes* abundance and *Firmicutes* to *Bacteroidota* ratio showed a trend of increasing, in turn, from mesotemperate to tropical. *Proteobacteria* showed a trend of reduction from HLJ to SS. *Desulfobacterota* was mainly enriched in HLJ and SD and significantly different from those in HK and SS. *Unclassified_Bacteria*, *Verrucomicrobiota*, *Patescibacteria*, *Chloroflexi*, and *Actinobacteriota* were particularly high in the SS, while *Campylobacterota* was mostly enriched in the HLJ.

In addition, a total of 27 genera could be potential biomarkers to distinguish the climate zone where cattle were living. *c__Saccharimonadia*, *f__Christensenellaceae*, *f__Ruminococcaceae*, *f__Saccharimonadaceae*, *f__unclassified_Clostridia_UCG_014*, *g__Candidatus_Saccharimonas*, *g__Christensenellaceae_R_7_group*, *g__unclassified_Clostridia_UCG_014*, *o__Christensenellales*, *o__Clostridia_UCG_014*, *o__Coriobacteriales*, *o__RF39*, *o__Saccharimonadales*, *p__Firmicutes*, *p__Patescibacteria*, *s__unclassified_Christensenellaceae_R_7_group*, and *s__unclassified_Clostridia_UCG_014* were progressively enriched from the mesotemperate to the tropical, while the relative proportion of *c__Gammaproteobacteria*, *f__Pseudomonadaceae*, *g__Pseudomonas*, *p__Proteobacteria*, and *s__unclassified_Pseudomonas* were gradually decreased. Meanwhile, *f__Planococcaceae*, *g__Psychrobacter*, *g__Solibacillus*, *s__unclassified_Psychrobacter,* and *s__unclassified_Solibacillus* were only located in the mesotemperate (HLJ) and warm temperate (SD) regions and not in subtropical (HK) and tropical (SS) regions.

### 3.6. Functional Differences in Microbiota in Different Geographical and Climatic Areas

**Figure 4:**
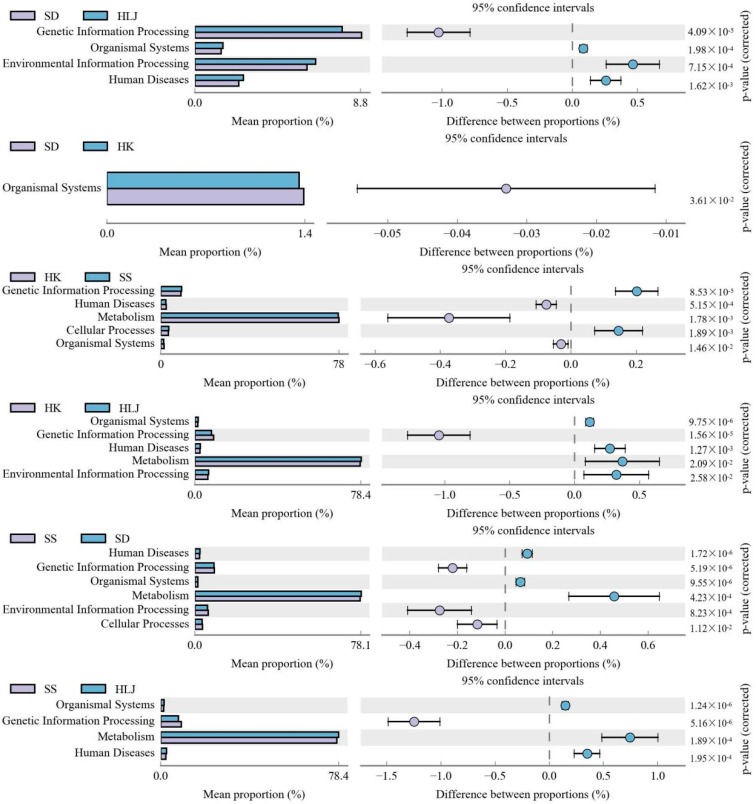
Fecal microbiome functions between comparisons through the enriched KEGG metabolic pathways.

A total of 44 Kyoto Encyclopedia of Genes and Genomes (KEGG) metabolic pathways were detected in the first hierarchy. Then, comparisons in pairs between the four geographical groups illustrated that six metabolic pathways were significantly different in all six pairs (Figure 4). Microbial functional comparison between two adjacent locations showed that the organismal system was successively decreased in abundance from HLJ, SD, and HK to SS. The enrichment of the other five functional genes was similar in HK and SD. When SD and HK were regarded as a whole, a longer distance comparison showed that metabolism and human diseases were more enriched in HLJ than in HK, and SD was more enriched than SS. Meanwhile, genetic information processing was higher in HK than in HLJ, and SS was higher than in SD. These comparisons were further verified by the analysis between HLJ and SS; in other words, the functional abundances of organismal system, metabolism, and human disease gradually decreased from mesotemperate to tropical, while that of genetic information processing significantly increased.

**Figure 5:**
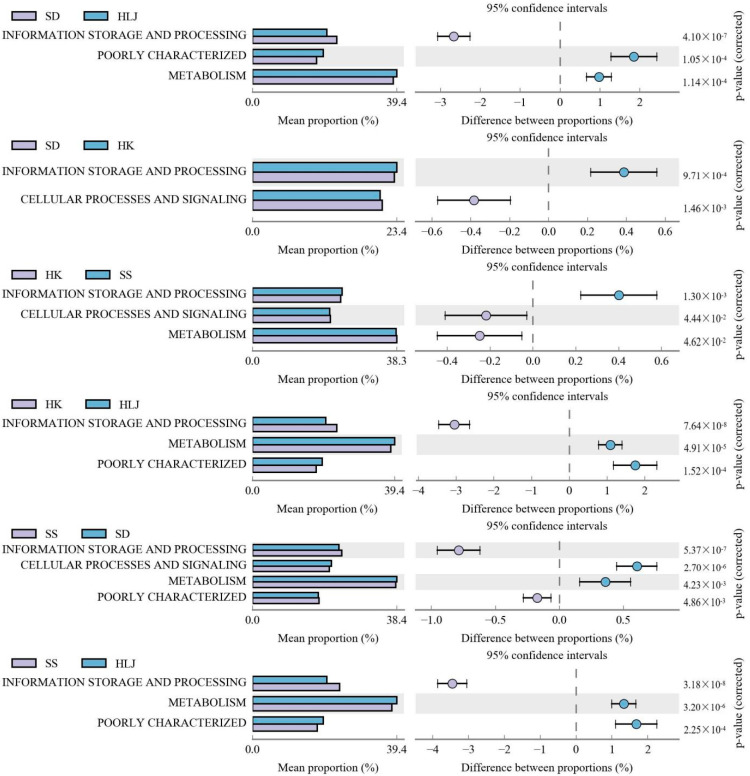
Fecal microbiome functions between comparisons with the COG functions analysis.

Compared with COG (clusters of orthologous groups) of proteins via group pairs, there were 4 metabolic pathways of protein function to be identified (Figure 5). The mean proportion of information storage and processing increased in sequence from HLJ, SD, and HK to SS. The relative abundance of metabolism in SD did not show a significant difference from that in HK. However, HLJ was more enriched than those in SD and HK, which were higher than in SS. No significant difference was found in cellular processes and signaling in HLJ and SD, but they were obviously decreased in HK and SS, and the latter was more significant than the former.

### 3.7. Some Microbial Phenotypes at the Organism Level Were Matched to the Geographical and Climatic Regions

**Figure 6:**
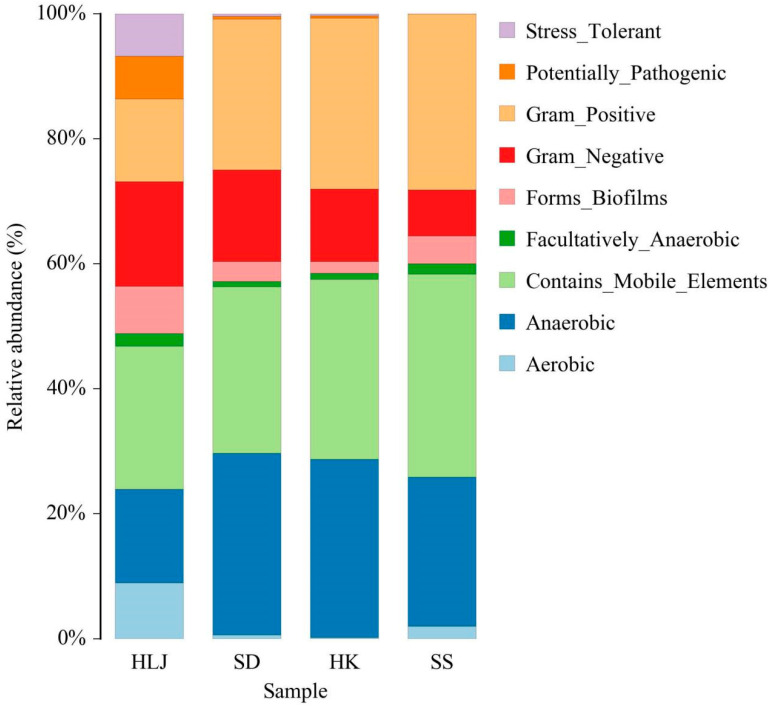
Differences in microbiota phenotype among different sampled locations.

Through pairwise comparison among the four geographical groups, nine discrepant phenotypes were identified at the microbial level (Figure 6 and Figure S6). From HLJ to SS, in turn, the Gram-positive was increased significantly, while the Gram-negative was decreased. Moreover, stress tolerant and potentially pathogenic were mainly isolated from HLJ and only found at low levels in the other places. The relative abundance of aerobic and forming of biofilms gradually decreased in the order of HLJ, SD, and HK and then rebounded significantly in SS.

## 4. Discussion

The gut microbiome of cattle plays an important role in facilitating the development of gut tissue and the immune system and then optimizing health and production efficiency [28]. The microbiota disorder in the gastrointestinal tract also contributes to the development of gas (such as methane, hydrogen, and CO_2_). Gases produced in excess can result in gaseous distension, which exerts pressure on the nearby organs, causing edema, pain, organ failure, and even death [29]. Previous findings suggested that acute and short-term heat stress environments resulted in the alteration of microbial activities, thus affecting the metabolism, endocrine pathways, adaptive immunity, and potential diseases in cows [11]. To the best of our knowledge, this is the first study to compare adaptive changes in the fecal microbiome of cattle from temperate to tropical climates and seek a microbial target that had a durable response to a high-temperature environment.

The bovine fecal microbiota is complex, and its composition can be influenced by numerous factors, such as age, diet, management system, stress, pathogeny, and climatic factors from the geographical location [14,30,31,32]. Our study showed that the diversity of fecal microbiota in the same location was undifferentiated among cattle of different ages, contrary to previous studies that reached a consensus that age is the main driving force in the establishment of microbial communities in calves [33,34]. Some researchers have reported that calf fecal communities begin to have consistency or reach maturity after approximately 3 months of age [30,35]. This divergence was likely because the young animals sampled in the current study were relatively more mature (6–12 months old) than those in previous studies. Of course, the limitation of a small sample size might also affect the significance of the difference, to which more attention should be given in future studies.

Consistent with previous studies, *Firmicutes* and *Bacteroidota* were the two most dominant phyla, the former representing 42.40–77.37% of all sequences, while the latter accounted for 10.89–30.22%. *Proteobacteria* (0.28–26.89%) ranked third [35,36]. Although the microbial diversity was not perfectly consistent with the geographical location via the analysis of alpha diversity (data not shown), beta diversity comparison uncovered the intuitive association of fecal microbiota composition with the climate progression from the north (cold) to the south (hot). It was demonstrated that *Firmicutes* showed a continuous increase from the mesotemperate to the tropical, accompanied by a rising *Firmicutes* to *Bacteroidota* ratio, but not a decrease in the *Bacteroidota* proportion. *Firmicutes* play a critical role in the microbial ecology of the bovine gut, and its relatively higher abundance in cattle suggests the possibility of it being more efficient in utilizing nutrients [31,32]. Díaz Carrasco et al. suggested that an increased *Firmicutes* to *Bacteroidota* ratio contributed to improved performance in bovines, including body weight gain and milk fat yield [37,38,39]. Restrictions of production performance are frequently observed during heat stress but are conquered in the tropics, probably through the adaptive or evolutionary regulation of the *Firmicutes* to *Bacteroidota* ratio of the gut microbiome. Previous research showed that heat stress-induced sudden death was mainly because of heart failure and its immediate insufficient supply of blood oxygen [40,41]. However, the *Firmicutes* to *Bacteroidota* ratio can also influence cardiorespiratory fitness. Evidence in healthy young adults suggested that maximal oxygen consumption and a highly efficient energy harvest were positively associated with the ratio [42]. Unlike other studies, a regular decrease in *Bacteroidota* abundance was not found at the same time in the present study [18,31,43], which was possibly due to the influence of breed, diet, management, and so on. Moreover, as in a previous report, *Proteobacteria* was ranked third or fourth among these predominant microflora in the gastrointestinal tract but showed a clear decreasing trend from HLJ to SS [29]. Hence, *Proteobacteria* might be a marker in the level of phylum.

*Desulfobacterota* is a sulfate reducer and is present in cold habitats. Our results showed that *Desulfobacterota* was more enriched in HLJ and SD than in HK and SS. This may be related to the greater enrichment of sulfur in the sampled northern areas and the preference of cold environments for spore formation [44]. The *Verrucomicrobia* phylum is known to possess the ability to degrade mucin, thus destroying intestinal barrier integrity and promoting the penetration of pathogenic microbes [45]. *Proteobacteria,* which are Gram-negative bacteria, are often used as indicators of pathogenicity, inflammation, and gut dysbiosis caused by diet changes or enrichment of stress-response genes [46,47,48]. An increase in their abundance is associated with several diseases that sometimes occur in humans. Therefore, an environment with extreme temperature (heat or cold) stress may contribute to the disruption of the cow’s gut microbiota homeostasis or alteration of zoonotic disease risk. Interestingly, *Proteobacteria* was also reported to be enriched mostly in humid and arid samples, thus possibly implying a beneficial change for environmental adaptation [49]. More definitive conclusions require further study. *Patescibacteria* can potentially perform fermentative recycling of organic carbon [50]. Although little information has been found in the animal gut, *Chloroflexi* arises under anaerobic conditions during rice growth to normalize and regulate the soil bacterial composition based on their oxygen requirements, resulting in stable bacterial communities [51]. *Actinobacteriota* abundance is always higher in arid regions [49]; the three phyla mentioned above were particularly highest in the SS and judged to have been vital strategies for adaptation to tropical high temperatures. *Campylobacterota,* mostly enriched in the HLJ, is regarded as an evolutionary heat-preservation countermeasure to low temperatures because of its usual link with gastrointestinal health and its high presence in the obese population, such as overweight adolescents in Sweden [52,53].

At the genus level, 27 genera were selected to distinguish the climate zones of cattle. Most of them could be categorized into the phylum discussed above and provide more accurate flora targets with the ability to improve the heat resistance of cattle. For instance, *f__Ruminococcaceae* belongs to the phylum *Firmicutes* and was progressively enriched from the mesotemperate to the tropical climates. The genera was reported to have a higher abundance in the feces of calves fed pasteurized milk and acidified milk and could improve the fiber utilization ability of the gastrointestinal tract. It was because that *f__Ruminococcaceae*, as the first order cellulolytic bacteria, contributed to most of the xylanase and endoglucanase activities in the gastrointestinal tract, and then to degrade the cellulose into smaller oligo/disaccharides, which are then acted upon by other organisms [29]. These again confirmed the important role of *Firmicutes* in assimilating nutrients more efficiently under a high-temperature environment [54]. Our findings that the abundance of two representatives of *Proteobacteria*, *c__Gammaproteobacteria*, and *f__Pseudomonadaceae,* gradually decreased from the cold north to the hot south may also be a more precise and positive mechanism among cattle to adapt to living in a high-temperature environment. As mentioned earlier regarding *Proteobacteria*, dysbiosis with a preponderance of *Gammaproteobacteria* and its constituent family *Pseudomonadaceae* is associated with adverse outcomes, including necrotizing enterocolitis, late-onset sepsis, and developmental delay [55]. Downregulation of the two genera was presumed to be an active indication of the flora favorable for cattle to live in a tropical climate. In addition, there were four genera (namely, *f__Planococcaceae*, *g__Psychrobacter*, *g__Solibacillus*, *s__unclassified_Psychrobacter* and *s__unclassified_Solibacillus*) that were only located in the mesotemperate (HLJ) and warm temperate (SD) regions and not in subtropical (HK) and tropical (SS) regions, which possibly shared a similar mechanism with *Gammaproteobacteria*. These flora might not adapt to high-temperature stimulation at all.

With a continuous rise in temperature in the living environment, phenotypic variation in fecal microorganisms was observed and characterized by an increase in Gram-positive and a decrease in Gram-negative, aerobic, and forming of_biofilms. Gram-positive bacteria possess thicker cell walls and thus greater heat resistance than Gram-negative bacteria. Therefore, more Gram-positive bacteria were present in the gut of cattle in the tropical region. Similar to the increase in strict anaerobes and obligate anaerobes in the large intestine and small intestine under hypobaric hypoxia for 30 days [56], we found that long-term living in a tropical climate upregulated the aerobic phenotypic abundance, implying that oxygen was abundant not only for body metabolism of cattle but also for intestinal flora. Bacterial colonization or infection on surfaces such as intestinal mucosa are associated with biofilm formation. Bacterial biofilms are also known to increase resistance to antibiotics, antimicrobials, host immune responses, and so on [57,58]. The increased formation of biofilms may introduce a particular cold/heat tolerance to the colonized bacteria in the gut of cattle in HLJ and SS. Consistent with the phenotypic changes, the decreased functional abundances of the organismal system and metabolism from HLJ to SS illustrated an active adaptive response to the reduction in the supply of oxygen, materials, and energy, resulting from the increased consumption by vital organs for survival in a hot environment. Previous evidence suggested that the heat tolerance increase of gut flora was connected to the suppression of oxidative metabolism [59]. The increase in genetic information processing and information storage and processing sheds light on the exact mechanism by which gut flora deal with extremely hot conditions. Cellular processes and signaling may be pivotal in both cold and hot situations.

Two limitations of this study are the difference in cattle breeds and three or fewer cattle samples in each age of cattle from the same location, which may affect the analysis of the results to a certain extent. In addition, except for climatic temperature factors, other variables, such as diet, management, and humidity, were difficult to exclude. Therefore, future research should focus on tropical cattle breeds to collect more samples for analysis. Despite all this, this is still an important scientific direction to investigate how the gut microbiota responds to a high-temperature environment and then influences the heat tolerance of animals.

## 5. Conclusions

The present study showed clear differences in the fecal microbiomes of cattle from mesotemperate to tropical climates. With increasing temperatures, the abundance of *Firmicutes* increased, accompanied by an increasing *Firmicutes* to *Bacteroidota* ratio. *The content of Proteobacteria decreased continuously* from HLJ to SS. *Patescibacteria*, *Chloroflexi*, and *Actinobacteriota* may also be great strategies for adapting to tropical environments. The enrichment of different microbes in a high-temperature environment changed the flora phenotype, characterized by an increase in Gram-positive and a decrease in Gram-negative, aerobic, and forming_biofilms. Consistently, to address extremely hot conditions, the functional abundances of organismal systems and metabolism were decreased, and genetic information processing and information storage and processing were increased. The present study revealed the differences in the structure and function of the gut microbes of cattle from mesotemperate to tropical climates and provided an important reference for future research on the mechanism of heat tolerance regulated by the gut microbiota and a potential microbiota-based target to alleviate heat stress.

## Figures and Tables

**Table 1:** The total number of fecal samples collected, stratified for area and cattle age.

Age Samples No. Location	HLJ	SD	HK	SS
**4–6 years old (A)**	3	3	3	3
**1.5–2 years old (B)**	3	3	3	1
**6 months to 1 year old** **(C)**	3	3	3	3

Notes: HLJ, Daqing city in Heilongjiang province, China. SD, Qingdao city in Shandong province, China. HK, Haikou city in Hainan province, China. SS, Sansha city in Hainan province, China.

## Data Availability

Data related to this study can be found in the Tables S2 and S3 of Supplementary Materials.

## Supplementary Materials

The following supporting information can be downloaded at: https://www.mdpi.com/article/10.3390/microorganisms10081672/s1, Figure S1. Distribution diagram of feature number of each sample. Figure S2. The change profile of the fecal microbiota at the phylum level with increasing age. Data are expressed as mean ± standard deviation. Figure S3. The change profile of the fecal microbiota at the genus level with increasing age. Data are expressed as mean ± standard deviation. Figure S4. The *Firmicutes* to *Bacteroidota* ratio in fecal from four isolations. ** *p* < 0.01 vs. those in HLJ, SD and HK. Figure S5. Selected fecal microbiota biomarkers to distinguish the climate zone. Figure S6. Differences of microbiota phenotype in pairwise comparison; Table S1: The processing result statistics of samples sequencing data. Table S2: All abundance of all samples. Table S3: All reabundance of all samples.

## References

[1] Carvajal M.A., Alaniz A.J., Gutiérrez-Gómez C., Vergara P.M., Sejian V., Bozinovic F. (2021). Increasing importance of heat stress for cattle farming under future global climate scenarios. Sci. Total Environ..

[2] De Rensis F., Marconi P., Capelli T., Gatti F., Facciolongo F., Franzini S., Scaramuzzi R.J. (2002). Fertility in postpartum dairy cows in winter or summer following estrus synchronization and fixed time AI after the induction of an LH surge with GnRH or hCG. Theriogenology.

[3] Mishra S.R. (2020). Significance of molecular chaperones and micro RNAs in acquisition of thermo-tolerance in dairy cattle. Anim. Biotechnol..

[4] Wang Y., Yang C., Elsheikh N.A.H., Li C., Yang F., Wang G., Li L. (2019). HO-1 reduces heat stress-induced apoptosis in bovine granulosa cells by suppressing oxidative stress. Aging.

[5] Correia Sales G.F., Carvalho B.F., Schwan R.F., de Figueiredo Vilela L., Moreno Meneses J.A., Gionbelli M.P., da Silva Ávila C.L. (2021). Heat stress influence the microbiota and organic acids concentration in beef cattle rumen. J. Therm. Biol..

[6] Mishra S.R. (2021). Behavioural, physiological, neuro-endocrine and molecular responses of cattle against heat stress: An updated review. Trop. Anim. Health Prod..

[7] Lu X., Arbab A.A.I., Zhang Z., Fan Y., Han Z., Gao Q., Sun Y., Yang Z. (2020). Comparative Transcriptomic Analysis of the Pituitary Gland between Cattle Breeds Differing in Growth: Yunling Cattle and Leiqiong Cattle. Animals.

[8] Kong L., Liu G., Deng M., Lian Z., Han Y., Sun B., Guo Y., Liu D., Li Y. (2020). Growth retardation-responsive analysis of mRNAs and long noncoding RNAs in the liver tissue of Leiqiong cattle. Sci. Rep..

[9] Ahmad M., Bhatti J.A., Abdullah M., Javed K., Ali M., Rashid G., Uddin R., Badini A.H., Jehan M. (2018). Effect of ambient management interventions on the production and physiological performance of lactating Sahiwal cattle during hot dry summer. Trop. Anim. Health Prod..

[10] Chen S., Wang J., Peng D., Li G., Chen J., Gu X. (2018). Exposure to heat-stress environment affects the physiology, circulation levels of cytokines, and microbiome in dairy cows. Sci. Rep..

[11] Planer J.D., Peng Y., Kau A.L., Blanton L.V., Ndao I.M., Tarr P.I., Warner B.B., Gordon J.I. (2016). Development of the gut microbiota and mucosal IgA responses in twins and gnotobiotic mice. Nature.

[12] Lee W.-J., Hase K. (2014). Gut microbiota–generated metabolites in animal health and disease. Nat. Chem. Biol..

[13] Zhao L., Li X., Atwill E.R., Aly S.S., Williams D.R., Su Z. (2022). Dynamic changes in fecal bacterial microbiota of dairy cattle across the production line. BMC Microbiol..

[14] Gabanyi I., Lepousez G., Wheeler R., Vieites-Prado A., Nissant A., Wagner S., Moigneu C., Dulauroy S., Hicham S., Polomack B. (2022). Bacterial sensing via neuronal Nod2 regulates appetite and body temperature. Science.

[15] Louca P., Nogal A., Wells P.M., Asnicar F., Wolf J., Steves C.J., Spector T.D., Segata N., Berry S.E., Valdes A.M. (2021). Gut microbiome diversity and composition is associated with hypertension in women. J. Hypertens..

[16] Lan D., Jiangjiang Z., Lin B., Chen Y., Huang C., Xiong X., Fu M., Mipam T.D., Ai Y., Zeng B. (2017). Correlations between gut microbiota community structures of Tibetans and geography. Sci. Rep..

[17] Zhao J., Yao Y., Li D., Xu H., Wu J., Wen A., Xie M., Ni Q., Zhang M., Peng G. (2018). Characterization of the Gut Microbiota in Six Geographical Populations of Chinese Rhesus Macaques (Macaca mulatta), Implying an Adaptation to High-Altitude Environment. Microb. Ecol..

[18] Goel A., Kim B.-J., Ncho C.-M., Jeong C.-M., Gupta V., Jung J.-Y., Ha S.-Y., Lee D.-H., Yang J.-K., Choi Y.-H. (2021). Dietary Supplementation of Shredded, Steam-Exploded Pine Particles Decreases Pathogenic Microbes in the Cecum of Acute Heat-Stressed Broilers. Animals.

[19] Yang Z., Yang J., Zhou M., Yin P., Chen Z., Zhao Q., Hu K., Liu Q., Ou C.-Q. (2021). Hourly temperature variability and mortality in 31 major Chinese cities: Effect modification by individual characteristics, season and temperature zone. Environ. Int..

[20] Ding D., Zhu J., Gao Y., Yang F., Ma Y., Cheng X., Li J., Dong P., Yang H., Chen S. (2022). Effect of cattle farm exposure on oropharyngeal and gut microbial communities and antibiotic resistance genes in workers. Sci. Total Environ..

[21] Bolyen E., Rideout J.R., Dillon M.R., Bokulich N.A., Abnet C.C., Al-Ghalith G.A., Alexander H., Alm E.J., Arumugam M., Asnicar F. (2019). Reproducible, interactive, scalable and extensible microbiome data science using QIIME 2. Nat. Biotechnol..

[22] Callahan B.J., Mcmurdie P.J., Rosen M.J., Han A.W., Johnson A.J., Holmes S.P. (2016). DADA2: High-resolution sample inference from Illumina amplicon data. Nat. Methods.

[23] Quast C., Pruesse E., Yilmaz P., Gerken J., Schweer T., Yarza P., Peplies J., Glöckner F.O. (2013). The SILVA ribosomal RNA gene database project: Improved data processing and web-based tools. Nucleic Acids Res..

[24] Lozupone C., Lladser M.E., Knights D., Stombaugh J., Knight R. (2011). UniFrac: An effective distance metric for microbial community comparison. ISME J..

[25] Segata N., Izard J., Waldron L., Gevers D., Miropolsky L., Garrett W.S., Huttenhower C. (2011). Metagenomic biomarker discovery and explanation. Genome Biol..

[26] Parks D.H., Tyson G.W., Hugenholtz P., Beiko R.G. (2014). STAMP: Statistical analysis of taxonomic and functional profiles. Bioinformatics.

[27] Ward T., Larson J., Meulemans J., Hillmann B., Lynch J., Sidiropoulos D., Spear J.R., Caporaso G., Blekhman R., Knight R. (2017). BugBase predicts organism-level microbiome phenotypes. bioRxiv.

[28] MacPherson A.J., Harris N.L. (2004). Interactions between commensal intestinal bacteria and the immune system. Nat. Rev. Immunol..

[29] Xu Q., Qiao Q., Gao Y., Hou J., Hu M., Du Y., Zhao K., Li X. (2021). Gut Microbiota and Their Role in Health and Metabolic Disease of Dairy Cow. Front. Nutr..

[30] Li M., Wang Z., Wang L., Xue B., Hu R., Zou H., Liu S., Shah A.M., Peng Q. (2022). Comparison of changes in fecal microbiota of calves with and without dam. PeerJ.

[31] Corrêa P.S., Jimenez C.R., Mendes L.W., Rymer C., Ray P., Gerdes L., da Silva V.O., De Nadai Fernandes E.A., Abdalla A.L., Louvandini H. (2021). Taxonomy and Functional Diversity in the Fecal Microbiome of Beef Cattle Reared in Brazilian Traditional and Semi-Intensive Production Systems. Front. Microbiol..

[32] Redding L.E., Berry A.S., Indugu N., Huang E., Beiting D.P., Pitta D. (2021). Gut microbiota features associated with Clostridioides difficile colonization in dairy calves. PLoS ONE.

[33] Dill-McFarland K.A., Breaker J.D., Suen G. (2017). Microbial succession in the gastrointestinal tract of dairy cows from 2 weeks to first lactation. Sci. Rep..

[34] Dill-McFarland K.A., Weimer P.J., Breaker J.D., Suen G. (2019). Diet Influences Early Microbiota Development in Dairy Calves without Long-Term Impacts on Milk Production. Appl. Environ. Microbiol..

[35] Klein-Jöbstl D., Schornsteiner E., Mann E., Wagner M., Drillich M., Schmitz-Esser S. (2014). Pyrosequencing reveals diverse fecal mi-crobiota in Simmental calves during early development. Front. Microbiol..

[36] Meale S.J., Li S., Azevedo P., Derakhshani H., Plaizier J.C., Khafipour E., Steele M.A. (2016). Development of Ruminal and Fecal Micro-biomes Are Affected by Weaning but Not Weaning Strategy in Dairy Calves. Front. Microbiol..

[37] Díaz Carrasco J.M., Cabral C., Redondo L.M., Pin Viso N.D., Colombatto D., Farber M.D., Fernández Miyakawa M.E. (2017). Impact of Chestnut and Quebracho Tannins on Rumen Microbiota of Bovines. Biomed. Res. Int..

[38] Jami E., White B.A., Mizrahi I. (2014). Potential role of the bovine rumen microbiome in modulating milk composition and feed ef-ficiency. PLoS ONE.

[39] Meyer F., Paarmann D., D’Souza M., Olson R., Glass E.M., Kubal M., Paczian T., Rodriguez A., Stevens R., Wilke A. (2008). The met-agenomics RAST server—A public resource for the automatic phylogenetic and functional analysis of metagenomes. BMC Bioinformatics.

[40] Zhang X., Chen B., Wu J., Sha J., Yang B., Zhu J., Sun J., Hartung J., Bao E. (2020). Aspirin Enhances the Protection of Hsp90 from Heat-Stressed Injury in Cardiac Microvascular Endothelial Cells Through PI3K-Akt and PKM2 Pathways. Cells.

[41] Chen H., Adam A., Cheng Y., Tang S., Hartung J., Bao E. (2015). Localization and expression of heat shock protein 70 with rat myocardial cell damage induced by heat stress in vitro and in vivo. Mol. Med. Rep..

[42] Turnbaugh P.J., Ley R.E., Mahowald M.A., Magrini V., Mardis E.R., Gordon J.I. (2006). An Obesity-Associated Gut Microbiome with Increased Capacity for Energy Harvest. Nature.

[43] Li L., Zhao X. (2015). Comparative analyses of fecal microbiota in Tibetan and Chinese Han living at low or high altitude by barcoded 454 pyrosequencing. Sci. Rep..

[44] Watanabe M., Kojima H., Fukui M. (2013). *Desulfotomaculum intricatum* sp. nov., a sulfate reducer isolated from freshwater lake sediment. Int. J. Syst. Evol. Microbiol..

[45] Geerlings S.Y., Kostopoulos I., De Vos W.M., Belzer C. (2018). *Akkermansia muciniphila* in the Human Gastrointestinal Tract: When, Where, and How?. Microorganisms.

[46] Rizzatti G., Lopetuso L.R., Gibiino G., Binda C., Gasbarrini A. (2017). Proteobacteria: A Common Factor in Human Diseases. BioMed. Res. Int..

[47] Auffret M.D., Dewhurst R.J., Duthie C.-A., Rooke J.A., Wallace R.J., Freeman T.C., Stewart R., Watson M., Roehe R. (2017). The rumen microbiome as a reservoir of antimicrobial resistance and pathogenicity genes is directly affected by diet in beef cattle. Microbiome.

[48] Wu R., Mei X., Wang J., Sun W., Xue T., Lin C., Xu D. (2019). Zn(ii)-Curcumin supplementation alleviates gut dysbiosis and zinc dyshomeostasis during doxorubicin-induced cardiotoxicity in rats. Food Funct..

[49] Hernández-Álvarez C., García-Oliva F., Cruz-Ortega R., Romero M.F., Barajas H.R., Piñero D., Alcaraz L.D. (2021). Squash root microbiome transplants and metagenomic inspection for in situ arid adaptations. Sci. Total Environ..

[50] Dong X., Zhang C., Li W., Weng S., Song W., Li J., Wang Y. (2021). Functional diversity of microbial communities in inactive seafloor sulfide deposits. FEMS Microbiol. Ecol..

[51] Kikuchi H., Watanabe T., Jia Z., Kimura M., Asakawa S. (2007). Molecular analyses reveal stability of bacterial communities in bulk soil of a Japanese paddy field: Estimation by denaturing gradient gel electrophoresis of 16S rRNA genes amplified from DNA accompanied with RNA. Soil Sci. Plant Nutr..

[52] Man S.M. (2011). The clinical importance of emerging Campylobacter species. Nat. Rev. Gastroenterol. Hepatol..

[53] Zeigler C.C., Persson G.R., Wondimu B., Marcus C., Sobko T., Modéer T. (2012). Microbiota in the Oral Subgingival Biofilm Is Associated with Obesity in Adolescence. Obesity.

[54] Chen Y., Gao Y., Yin S., Zhang S., Wang L., Qu Y. (2020). Effect of acidified milk feeding on the intake, average daily gain and fecal microbiological diversity of Holstein dairy calves. Asian-Australas. J. Anim. Sci..

[55] Ho T.T.B., Groer M.W., Kane B., Yee A.L., Torres B.A., Gilbert J.A., Maheshwari A. (2018). Dichotomous development of the gut microbiome in preterm infants. Microbiome.

[56] Wang F., Zhang H., Xu T., Hu Y., Jiang Y. (2022). Acute exposure to simulated high-altitude hypoxia alters gut microbiota in mice. Arch. Microbiol..

[57] Drenkard E. (2003). Antimicrobial resistance of Pseudomonas aeruginosa biofilms. Microbes Infect..

[58] Jamal M., Ahmad W., Andleeb S., Jalil F., Imran M., Nawaz M.A., Hussain T., Ali M., Rafiq M., Kamil M.A. (2018). Bacterial biofilm and associated infections. J. Chin. Med. Assoc..

[59] Murray A.J., Montgomery H.E., Feelisch M., Grocott M.P.W., Martin D.S. (2018). Metabolic adjustment to high-altitude hypoxia: From genetic signals to physiological implications. Biochem. Soc. Trans..

