# Supplementary material for: The Association between Gut Microbiome Diversity and Composition and Heat Tolerance in Cattle
Source: Microorganisms. 2022 Aug 19;10(8):1672. doi: 10.3390/microorganisms10081672 (PMC9414853; doi:10.3390/microorganisms10081672)
Supplement: Supplementary file 1 [file microorganisms-10-01672-s001.zip › Figure legends for supplementary materials.pdf]

Figure legends for supplementary materials

Table S1 The processing result statistics of samples sequencing data.

Table S2 All abundance of all samples.

Table S3 All reabundance of all samples.

Figure S1. Distribution diagram of feature number of each sample.

Figure S2. The change profile of the fecal microbiota at the phylum level with increasing age. Data are expressed as mean ± standard deviation.

Figure S3. The change profile of the fecal microbiota at the genus level with increasing age. Data are expressed as mean ± standard deviation.

Figure S4. The *Firmicutes* to *Bacteroidota* ratio in fecal from four isolations. ** $P < 0.01$ vs. those in HLJ, SD and HK.

Figure S5. Selected fecal microbiota biomarkers to distinguish the climate zone.

Figure S6. Differences of microbiota phenotype in pairwise comparison.
