# Supplementary material for: The Association between Gut Microbiome Diversity and Composition and Heat Tolerance in Cattle
Source: Microorganisms. 2022 Aug 19;10(8):1672. doi: 10.3390/microorganisms10081672 (PMC9414853; doi:10.3390/microorganisms10081672)
Supplement: Supplementary file 1 [file microorganisms-10-01672-s001.zip › Table S1 The processing result statistics of samples sequencing data.pdf]

Table S1 The processing result statistics of samples sequencing data

Sample ID	Raw Reads	Clean Reads	Denoised Reads	Merged Reads	Nonchimeric Reads
HKA1	80,352	80,057	79,330	68,497	59,246
HKA2	80,218	79,950	79,390	70,851	64,870
HKA3	80,130	79,903	79,612	71,234	63,088
HKB1	80,069	79,819	79,452	69,552	61,996
HKB2	80,363	80,086	79,645	71,765	63,605
HKB3	79,717	79,458	79,070	69,304	62,135
HKC1	80,014	79,777	79,447	71,697	63,257
HKC2	80,150	79,887	79,607	71,242	62,403
HKC3	79,849	79,549	79,095	69,301	62,084
HLJA1	80,155	79,647	79,117	67,607	61,337
HLJA2	79,802	79,251	78,710	66,863	60,713
HLJA3	79,738	79,177	78,824	68,090	57,765
HLJB1	80,023	79,508	79,166	69,573	60,798
HLJB2	80,029	79,493	79,013	66,413	58,017
HLJB3	80,185	79,660	79,300	68,066	59,491
HLJC1	80,008	79,396	79,027	69,421	62,327
HLJC2	80,219	79,522	79,045	69,304	60,655
HLJC3	79,913	79,383	78,866	66,788	60,136
SDA1	80,120	79,626	79,208	65,132	60,153
SDA2	79,905	79,382	78,868	64,085	58,597
SDA3	80,128	79,582	79,038	65,099	58,902
SDB1	80,119	79,625	79,224	69,452	62,758
SDB2	79,771	79,267	78,830	66,395	59,267
SDB3	80,107	79,559	79,023	66,589	60,910
SDC1	80,272	79,716	79,334	68,471	60,290

Continued Table S1 The processing result statistics of samples sequencing data

Sample ID	Raw Reads	Clean Reads	Denoised Reads	Merged Reads	Nonchimeric Reads
SDC2	80,154	79,624	79,078	68,088	62,622
SDC3	80,271	79,734	79,283	65,513	58,467
SSA1	80,146	79,882	79,505	71,994	68,287
SSA2	79,924	79,702	79,225	70,640	66,530
SSA3	79,792	79,566	79,173	70,511	66,097
SSB1	79,877	79,638	79,340	70,586	67,229
SSC1	79,984	79,734	79,442	70,840	66,646
SSC2	80,139	79,846	79,480	70,971	67,734
SSC3	79,889	79,608	79,221	69,266	65,277
