# Supplementary figures and images for: The Association between Gut Microbiome Diversity and Composition and Heat Tolerance in Cattle
Source: Microorganisms. 2022 Aug 19;10(8):1672. doi: 10.3390/microorganisms10081672 (PMC9414853; doi:10.3390/microorganisms10081672)

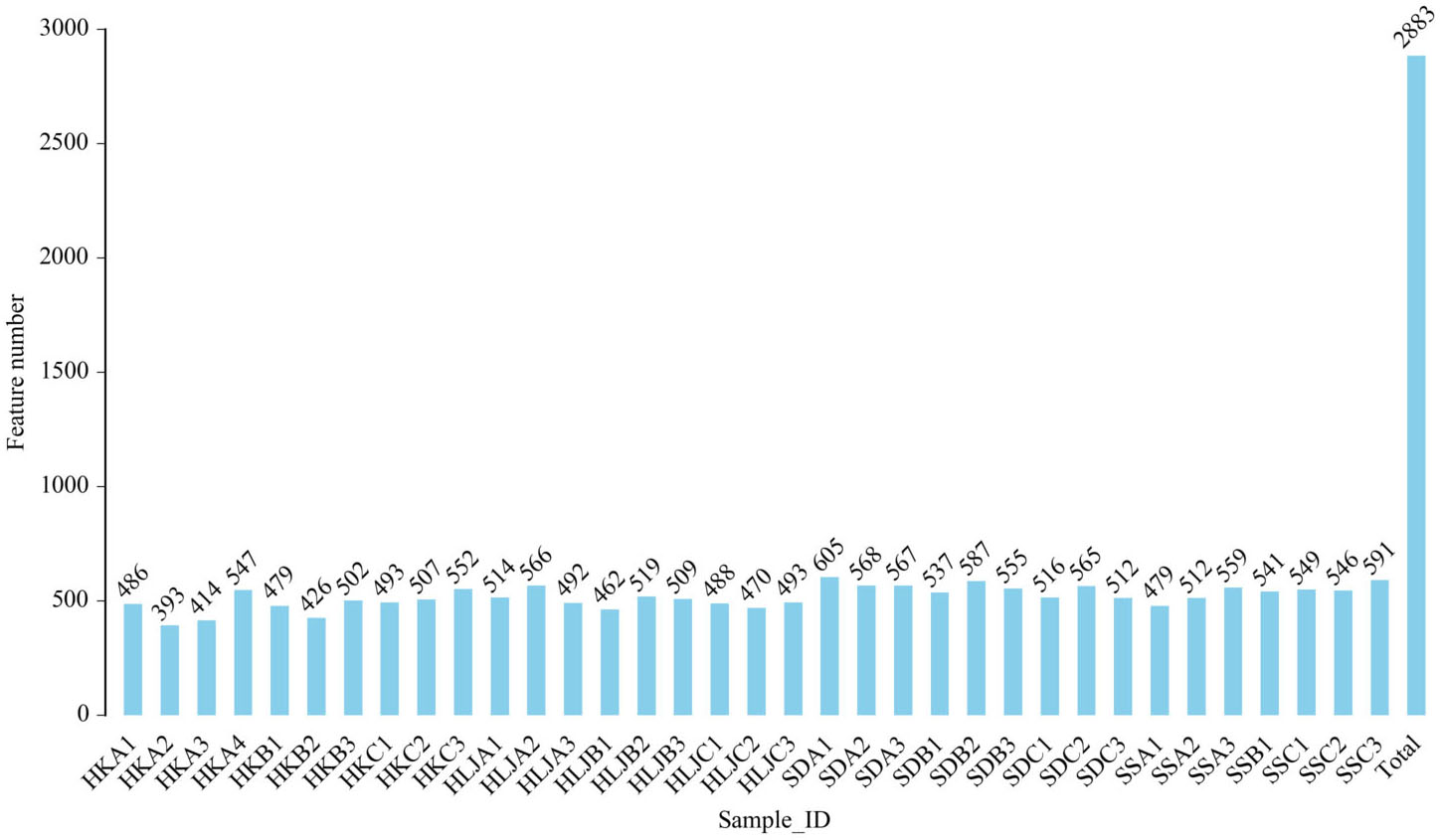

Supplement: Supplementary file 1 [file microorganisms-10-01672-s001.zip › Figure S1.jpg]

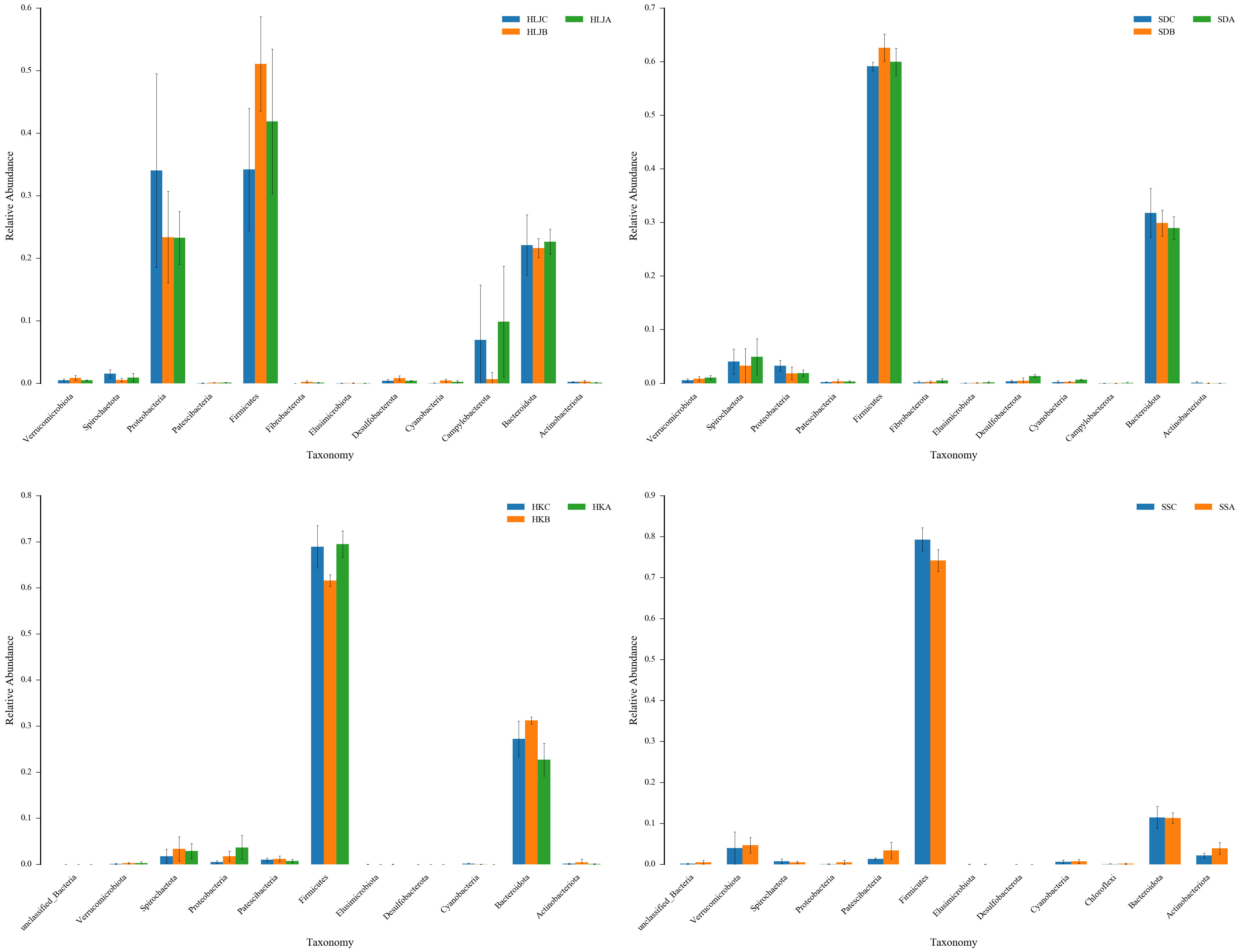

Supplement: Supplementary file 1 [file microorganisms-10-01672-s001.zip › Figure S2.jpg]

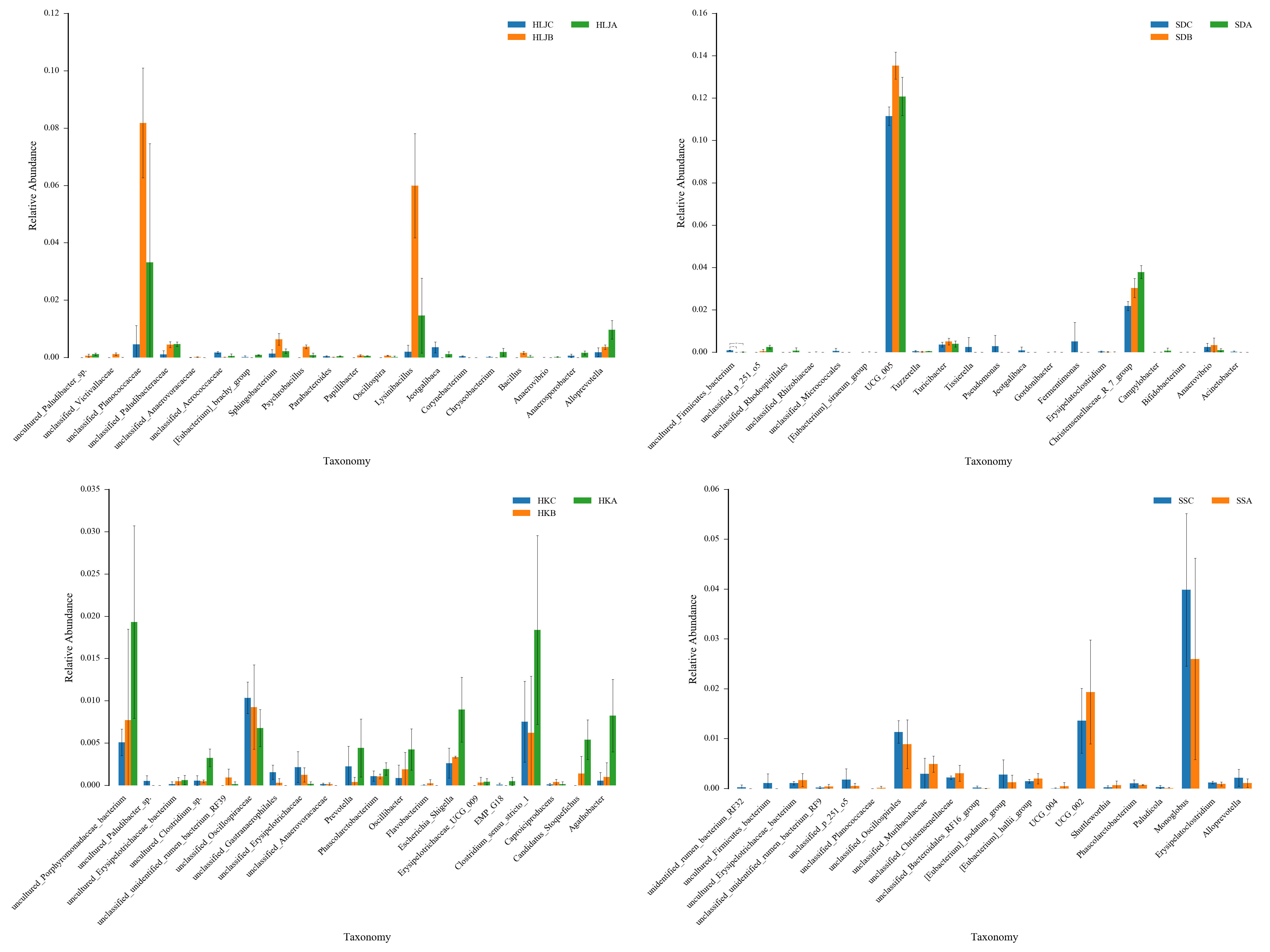

Supplement: Supplementary file 1 [file microorganisms-10-01672-s001.zip › Figure S3.jpg]

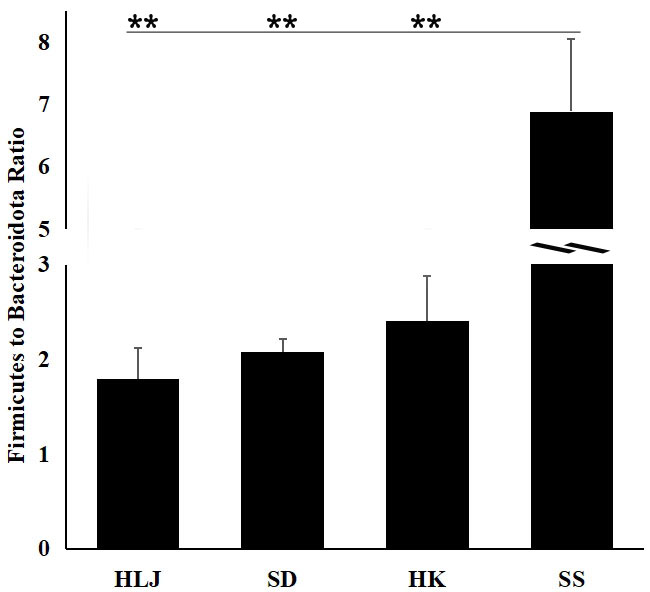

Supplement: Supplementary file 1 [file microorganisms-10-01672-s001.zip › Figure S4.jpg]

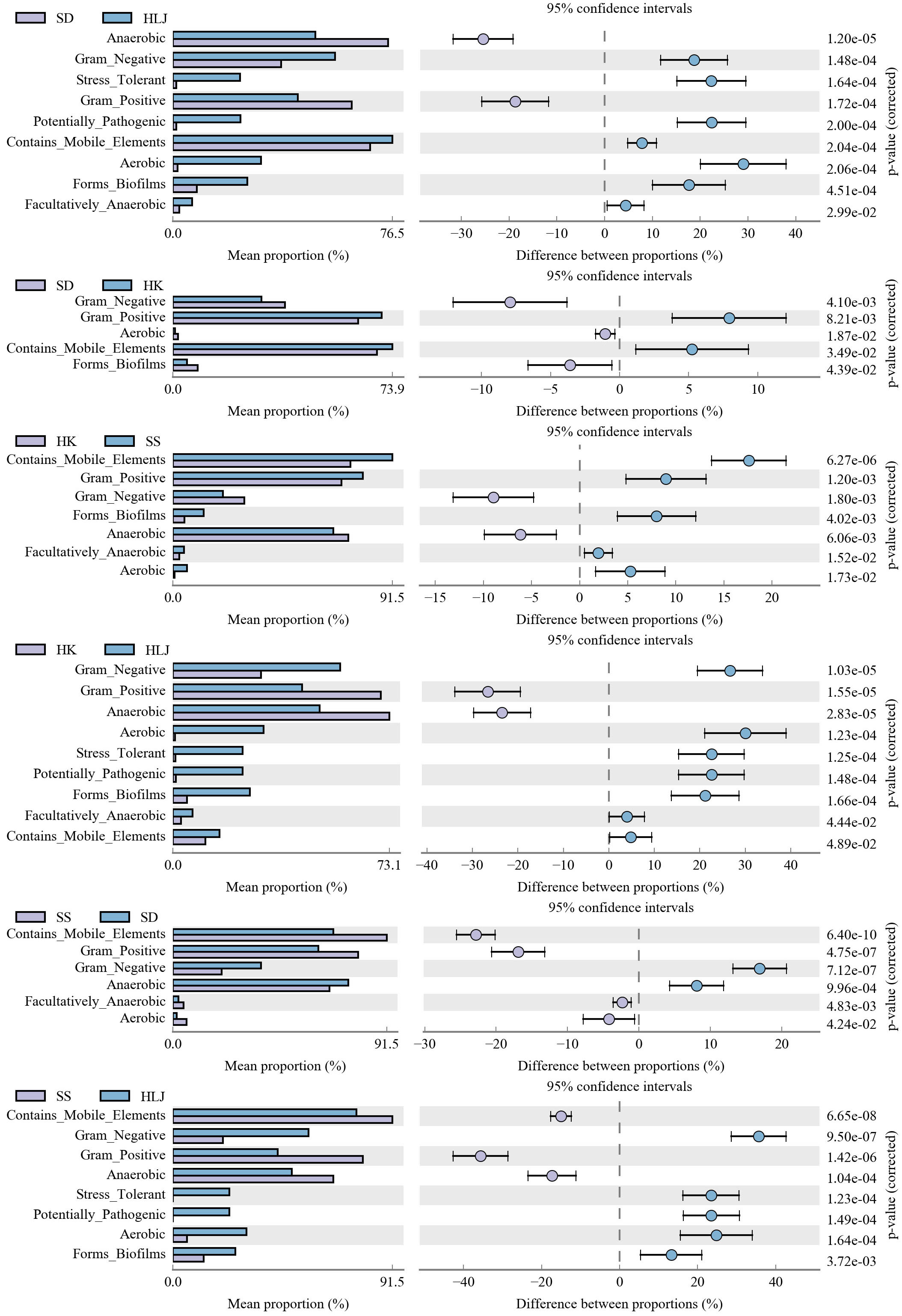

Supplement: Supplementary file 1 [file microorganisms-10-01672-s001.zip › Figure S6.jpg]
